# Comparison of clinical symptoms and bioimpedance to pulmonary capillary wedge pressure in heart failure

**DOI:** 10.1016/j.ahjo.2022.100133

**Published:** 2022-04-20

**Authors:** Monica Polcz, Jessica Huston, Meghan Breed, Marisa Case, Philip Leisy, Jeffrey Schmeckpeper, Lexie Vaughn, Jenna Helmer Sobey, Colleen Brophy, JoAnn Lindenfeld, Kyle Hocking, Bret Alvis

**Affiliations:** a Department of Surgery, Vanderbilt University Medical Center, Nashville, TN, USA; b Department of Medicine, Division of Cardiology, University of Pittsburgh Medical Center, Pittsburgh, PA, USA; c Department of Emergency Medicine, Vanderbilt University Medical Center, Nashville, TN, USA; d Department of Anesthesiology, Vanderbilt University Medical Center, Nashville, TN, USA; e Department of Medicine, Division of Cardiology, Vanderbilt University Medical Center, Nashville, TN, USA; f Department of Biomedical Engineering, Vanderbilt University, Nashville, TN, USA

**Keywords:** Congestion, Heart failure, Bioimpedance, PCWP, Readmission

## Abstract

**Introduction:**

Clinical symptoms of heart failure commonly include fatigue, edema, and shortness of breath. Unfortunately, clinical monitoring has proven unreliable in predicting congestion and the need for hospitalization. Biosensing wearables have been developed as a potential adjunct to clinical signs and symptoms to detect congestion before it becomes severe thus preventing a heart failure hospitalization.

**Hypothesis:**

Clinical signs and symptoms of heart failure will correlate with thoracic bioimpedance measurements (ZOE®) and pulmonary capillary wedge pressure (PCWP).

**Methods:**

One hundred and fifty-five subjects undergoing right heart catheterization (RHC) were prospectively enrolled. A Zo value (ohms) was obtained, jugular venous pressure (JVP) was estimated, edema graded, and shortness of breath (SOB) assessed in all subjects. RHC was performed by a scheduled cardiologist per routine. One-way ANOVA was performed to assess the relationship between variables. A Pearson correlation coefficient was used to compare the Zo value and PCWP.

**Results:**

Neither estimated JVP (cmH_2_O) (p = 0.65, n = 110) nor edema scores (p = 0.12, n = 110) demonstrated a significant relationship to PCWP. The presence of subjective SOB also did not demonstrate a significant association with PCWP (p = 0.99, n = 110). There was no correlation between ZOE® and PCWP (r = −0.08, p = 0.56, n = 56).

**Conclusions:**

These findings support the idea that traditional measures for monitoring heart failure patients are limited.

\

## 1 Introduction

Heart failure (HF) is a complex clinical syndrome that involves either impairment of ventricular filling or ejection of blood [1]. Clinical symptoms of heart failure commonly include fatigue, edema, and shortness of breath, but these often represent late manifestations of the disease [1], [2]. Heart failure symptoms significantly decrease health-related quality of life (HRQOL) and thus significant focus has been placed on preventing their onset [1], [3]. Accurately assessing heart failure severity before symptom onset is difficult and there is no single diagnostic test or data point that has proven reliable [1]. According to the ACCF/AHA Guidelines [4], volume status should be assessed at every patient encounter with serial assessment of weight, edema, jugular venous pressure (JVP), and orthopnea (SOB) [1]. Secondary analysis of data from the ESCAPE [5] trial demonstrated JVP as the only useful surrogate for predicting pulmonary capillary wedge pressure (PCWP) >22 mmHg [6], [7]. Orthopnea was only predictive of PCWP >28 mmHg [6], [7]. While clinical monitoring can detect current congestive symptoms, it is unreliable for predicting subclinical congestion and guiding therapeutic decisions to prevent acute decompensated heart failure and the need for hospital admission in patients with HF [8], [9], [10], [11], [12], [13].

Biosensing wearables have been developed as a potential adjunct to clinical symptoms for the assessment of heart failure, especially in the outpatient setting. Measurement of bioelectrical impedance (BI) is an example of such technology that multiple devices are based upon [14]. The ZOE® (Noninvasive Medical Technologies, Las Vegas, NV, USA) is a BI device cleared by the FDA in 2004 which measures the resistance of the thoracic cavity via a measurement of the time it takes a 100 kHz electric current to travel from the top to the bottom of the thorax [15], [16]. Resistance of tissue is altered by its fluid content, and the device outputs a Zo value that theoretically inversely correlates with the fluid content of the lungs [15]. Normal resistance has been determined to be between 19 and 30 Ω [15], [16]. To date, despite being used as a predicate device on multiple FDA 510 K applications/clearance, the ZOE® device has not been extensively studied in heart failure. It has demonstrated some success in correlating chest radiographic findings associated with pulmonary edema [16] but the relationship to important central hemodynamic parameters (i.e. PCWP) is limited [17], [18].

This report provides prospectively collected data examining the correlation between clinical signs and symptoms of heart failure using simultaneous bioimpedance measurements (ZOE®), and PCWP obtained in subjects with heart failure undergoing elective right heart catheterization (RHC) at a single large volume academic center.

## 2 Methods

This prospective, observational study was approved by the University of Alabama Birmingham Institutional Review Board through Vanderbilt University Medical Center Institutional Review Board. One hundred and fifty-five subjects undergoing elective RHC were approached for enrollment in the study. All subjects were positioned in the semi-recumbent position with the head of bed between 45 and 90 degrees and requested to remain still. ZOE® leads were placed per manufacturer's instructions, with hair shaved as needed for appropriate lead contact, and the Zo value (ohms) was obtained and recorded by one of the study personnel (MP, PL). Three values were obtained, one immediately after another, and averaged for each patient. Subjects were also questioned regarding subjective resting SOB and a focused physical exam was performed where edema grade was noted [19] and JVP was estimated [7], [20], [21], [22]. Demographic information and comorbidities were obtained from the medical record. RHC was performed by the scheduled interventional cardiologist per standard technique using central vein cannulation with a pulmonary artery catheter (PAC; Edwards Life Sciences Corporation, Irvine, CA, USA) inserted with the balloon tip inflated into the pulmonary artery and into “wedge” position. All waveforms were obtained at end expiration in spontaneously breathing patients and recorded to the electronic medical record. RHC tracings were then reviewed and PCWP values were assigned by a single board certified intensivist specialized (BA) in cardiovascular medicine who was blinded to Zo value and physical examination findings.

### 2.1 Statistical analysis

Clinical variables (JVP, edema grade, and subjective SOB) were evaluated for normality using a D'Agostino-Pearson normality test and then by one-way analysis of variance (ANOVA) with multiple comparisons across means of groups to observe the relationship between the dependent and independent variables. Pearson correlation coefficient between Zo value and PCWP was calculated using GraphPad Prism software (San Diego, CA). P-values less than 0.05 and a log worth of greater than 1 were considered significant. Multivariate analysis using linear regression on the variables the log worth and p-value were calculated in the ability to predict PCWP to compare all groups with principal component analysis to display as directional vectors by utilizing the eigenvectors of the covariance matrix. Sensitivity, specificity, positive and negative predictive values were also calculated using ROC curves for the ability of each clinical variable to predict PCWP >22 mmHg [6], [7].

## 3 Results

**Fig. 1:**
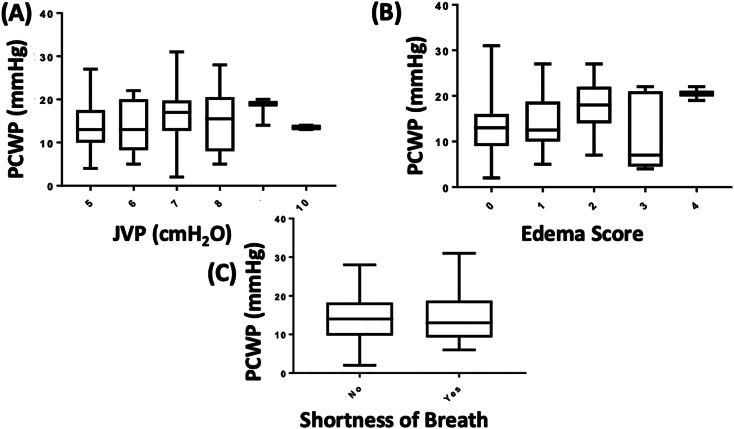
Box-and-Whisker Plot of Analysis of Variance (ANOVA) for Jugular Venous Pressure (JVP; A), Edema Score (B), and Shortness of Breath (SOB; C) to Pulmonary Capillary Wedge Pressure (PCWP) in heart failure patients. JVP measured at time of right heart catheterization demonstrated no statistically significance and no correlation (n = 110, p = 0.65, r = 0.17). Edema scores measured at time of right heart catheterization by study personnel demonstrated no statistically significance (P = 0.12) and no correlation (n = 110; R^2^ = 0.07). Whether a patient verbalized subjective shortness of breath at time of right heart catheterization by study personnel demonstrated no statistically significance (n = 110; P = 0.99) compared.

**Fig. 2:**
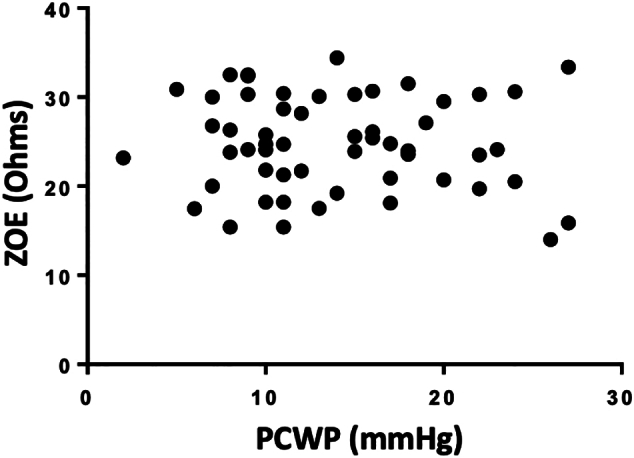
Pearson Correlation of ZOE® measurements compared to measured Pulmonary Capillary Wedge Pressure (PCWP) in heart failure patients. Relationship of bioimpendence as measured by the ZOE® device (Ohms) at time of right heart catheterization (RHC) demonstrated no correlation (n = 56. r = −0.08; P = 0.56).

Of the 155 enrolled, only 56 subjects had ZOE® measurements and 126 had interpretable PCWP tracings. Clinical signs and symptoms of congestion were obtained in 110 patients prior to right heart catheterization. Demographic information is listed in Table 1. Neither estimated JVP (cmH_2_O) (n = 110, p = 0.65; Fig. 1A) nor edema scores (n = 110, p = 0.12; Fig. 1B) demonstrated a significant relationship to PCWP [one way analysis of variance (ANOVA)]. The presence of subjective SOB also did not demonstrate significant association with PCWP (p = 0.99; n = 110 parametric Student's *t*-test, Fig. 1C). There was no correlation between ZOE® and PCWP (r = −0.08, n = 56, p = 0.56; Fig. 2).

**Table 1:** Demographic table of the outpatient RHC cohort. This table demonstrates the subject characteristics as median (iqr) or n(%). Abbreviations: BMI = body mass index, PAD = pulmonary artery diastolic pressure, PCWP = pulmonary capillary wedge pressure, CI = cardiac index, EF = ejection fraction, ASA = American Society of Anesthesiology, RHC = right heart catheterization, HF = heart failure.

	All	Male	Female
Age	56 (44–68)	58 (45–68)	53 (43–64)
Sex	69 (62.7%)		
BMI (kg/m^2^)	29.3 (25.7–33.5)	29.2 (25.8–33.2)	29.5 (25.8–34.7)
PAD (mmHg)	16 (11.5–22)	16 (10−22)	18 (13−22)
PCWP (mmHg)	13.5 (9.75–18.25)	14 (9–19)	13 (10–18)
PCWP > 22 mmHg	15 (14%)	12 (17%)	3 (7%)
CI (L/min/m^2^)	2.46 (2.06–2.87)	2.49 (2.12–2.85)	2.35 (1.80–2.87)
EF (%)			
<35	23 (21%)	18 (26.1%)	5 (12.2%)
35–45	7 (6.4%)	2 (2.9%)	5 (12.2%)
45–55	19 (17.3%)	10 (14.5%)	9 (22.0%)
>55	61 (55.5%)	39 (56.5%)	22 (53.6%)
ASA status			
1	0	0	0
2	4 (3.6%)	2 (2.9%)	2 (4.9%)
3	101 (91.8%)	64 (92.8%)	37 (90.2%)
4	5 (4.5%)	3 (4.3%)	2 (4.9%)
RHC (n; %)			
HF diagnostic evaluation	18 (16.3%)	5 (7.3%)	13 (31.7%)
HF maintenance care evaluation	30 (27.3%)	25 (36.2%)	5 (12.2%)
HF post-transplant graft evaluation	62 (56.4%)	39 (56.5%)	23 (56.1%)
TOTAL (n)	110	69	41

**Table 2:** Number of subjects with designated history and physical examination findings and the percentage (%) of those with measured PCWP>22 mmHg. Abbreviations: JVP = jugular venous pressure; PCWP = pulmonary capillary wedge pressure.

	N	PCWP > 22 mmHg, %
JVP		
<8	91	16
8–12	19	16
13–16	0	
>16	0	
Peripheral edema		
0	64	13
1	28	18
2	11	27
3	5	20
4	2	50
Dyspnea		
Yes	28	14
No	82	17
ZOE		
<20	11	27
20–30	31	10
>30	14	14

Clinical variables also did not demonstrate a reliable ability to predict PCWP >22 mmHg (Table 2 & Supplemental Table 1). JVP was measured less than 8 cmH_2_O in 91 out of 110 subjects (83%) and between 8 and 12 cmH_2_O in 19 (17%) subjects. 16% of patients in both groups (JVP < 8 cmH_2_O and 8–12 cmH_2_O) were found to have PCWP measurements >22 mmHg. Edema grade > 2 demonstrated a sensitivity of 28% and specificity of 86% in predicting PCWP >22 mmHg (Supplemental Table 1). JVP >8 demonstrated a sensitivity of 100% and specificity of 6% in predicting PCWP >22 mmHg. Subjective SOB demonstrated a sensitivity of 78% and specificity of 26% in predicting PCWP >22 mmHg. Zo value <20 demonstrated a sensitivity 33% and specificity of 80% in predicting a PCWP >22 mmHg (Supplemental Table 1). This table addresses the operating characteristics of clinical variables as a diagnostic tool for assessing PCWP >22 mmHg. None of the variables listed exhibited a balanced sensitivity and specificity for diagnosing this cutoff value, making them insensitive markers of a PCWP >22 mmHg. Finally, principal component analysis of the variables was conducted with PCWP to visualize the relation of the variables to one another. This sort of analysis is exploratory but can give insight to the underlying utility of variables in relation to one another. When this data was run in a stepwise regression to assess a multivariate approach to predicting PCWP >22 mmHg, no variables were included with either the stopping rule using minimum Bayesian Information Criterion. When a p-value threshold of 0.25 was used for the stepwise regression, edema was entered into the analysis and resulted in an insignificant nominal logistic fit to predicting PCWP >22 mmHg (p = 0.11, AUC = 0.60). Additionally, if all clinical signs and symptoms (JVP, edema, and dyspnea) were entered into a multivariate nominal logistic fit, edema displayed the greatest log worth in predicting PCWP >22 mmHg (Log worth = 0.9, p = 0.16) but none were statistically significant.

## 4 Discussion

The primary findings of this study are: 1) common clinical signs and symptoms, JVP, edema, and SOB did not correlate with measured PCWP values and 2) thoracic bioimpedance, ZOE®, measurements did not correlate to measured PCWP values in those same patients. These data provide a look at how these recommended examination findings and a common non-invasive measurement provide insight into a patient's PCWP, the gold standard for volume status [17].

History and physical (H&P) examination remains central in the management of patients with HF [7]. Despite the declining emphasis on the H&P, the use has routinely been highlighted in the management of HF and is used by clinicians to assess underlying hemodynamic state [6], [7]. The determination of a patient having an elevated PCWP is routinely based on JVP, SOB and edema [7]. In the ESCAPE trial [5], a multivariable model with PCWP ≥22 mmHg as the dependent variable was performed and demonstrated that only an elevated JVP, as defined as ≥12 mmHg, demonstrated the associated with elevated PCWP, defined as ≥22 mmHg (JVP odds ratio, 3.3; 95% CI, 1.8, 6.1) in stable advanced heart failure patients [6], [23]. SOB became associated with elevated PCWP once PCWP was measured ≥28 mmHg [6]. In our data set, the patient's with JVP elevated between 8 and 12 cmH_2_O only 16% had elevated PCWP >22 mmHg. Half of the patients with 4+ pitting edema had PCWP>22 mmHg and only 14% of the patients with SOB had elevated PCWP>22 mmHg. Of note, the subjects included in this study demonstrated a wider range of HF severity including a lower proportion of patients with PCWP>22 mmHg compared to the population investigated in the ESCAPE trial. The logistic regression of the variables conducted in Supplemental Table 1 resulted in nonsignificant results of using each variable independently as a diagnostic predictor of an elevated PCWP >22 mmHg.

Our findings are more consistent with published studies that conclude that signs and symptoms of JVP, edema and/or SOB have limitations and may not be as reliable in determining the actual intra-vascular volume in patients [24], [25]. While these data are not presented to disparage the importance of a cornerstone of patient care, the H&P, they are simply investigated to better appreciate their abilities and limitations when used in the care of HF patients.

**Supplemental Fig. 1:**
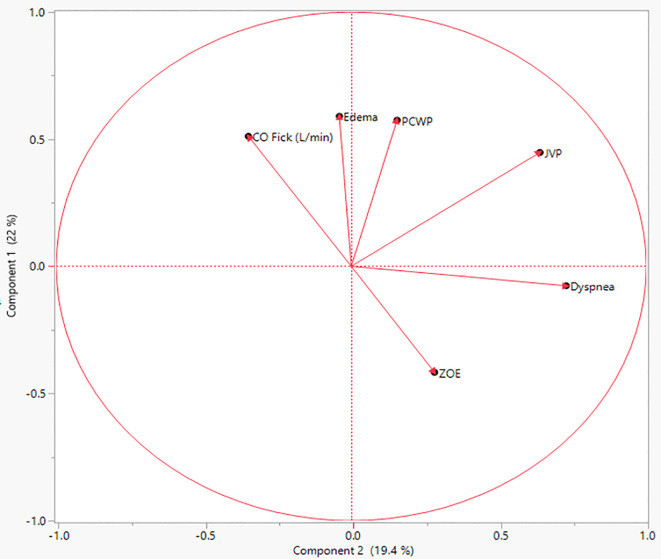
Principle component analysis of investigational variables demonstrating directional vectors by utilizing the eigenvectors of the covariance matrix. This analysis visually demonstrates the relationship of increasing values for each variable to one another. Abbreviations: PCWP = pulmonary capillary wedge pressure; JVP = jugular venous pressure; CO = cardiac output.

ZOE® has been used as the predicated device for multiple medical devices, specifically, for another thoracic impedance device ReDs (K150095). These data suggest that thoracic impedance is not an accurate measure of volume status as determined by PCWP. Interestingly, this point is visually clear in the principal component analysis demonstrating directional vectors of how increasing ZOE® measurements (less thoracic fluid) track along with the SOB findings (Supplemental Fig. 1). This does make clinical sense given what is measured. Thoracic bioimpedance measures the resistance (Ohms) of the thoracic region at 100 kHz. With a decrease in resistance representing “fluid” accumulation in the thoracic cavity, it stands to reason that decreasing ZOE® measurements would correlate to increasing SOB.

There were limitations of this study. As with any observational data there were traditional limitations such as: prone to bias and cannot be used to demonstrate causality. The three averaged ZOE® measurements may not have represented the best way of data capture to analyze its efficacy. While less practical, continuous measurements for larger sampling rates may provide more accurate data. Bioimpedance also has inherent limitations with measurement effect from body positioning, body tissue (muscle and adiposity) composition, presence of cutaneous hair or sweat, and lead placement. Of note, ZOE® measurements were taken at only one time point and only on patients that agreed to have the necessary areas shaved to allow proper electrode conductance limiting the number of enrolled patients who had ZOE® readings. Additionally, while 110 subjects provided usable PCWP tracings only 15 had PCWP >22 mmHg, potentially under powering the study.

## 5 Conclusion

Given the findings of this observational study, JVP, edema, and SOB appear to be unreliable in their ability to estimate the PCWP. JVP may be of some utility at extreme values. Furthermore, low enrollment rates for ZOE measurements highlighted the perceived discomfort and inconvenience of the bioimpedance electrodes and the need for more comfortable and patient focused methods for acquiring bioimpedance data to better study its utility. In this study, there was no correlation between thoracic bioimpedance, a non-invasive alternative method for investigating a patient's congestion status, and PCWP measured with a PAC. These findings support the idea that traditional measures for monitoring heart failure patients are limited and there is the significant need for reliable and accurate non-invasive volume status monitoring that can translate to both the hospital and the home setting.

The following are the supplementary data related to this article.

Supplemental Table 1 Utility of the Clinical Findings in Detecting PCWP >22 mmHg. Abbreviation: LR = likelihood ratio; OR = odds ratio; CI = confidence interval. Supplemental Table 1

## Funding

This work was supported by two grants from the 10.13039/100000002National Institutes of Health (BA: R01HL148244) and (KH: R44HL140669).

## CRediT authorship contribution statement

**Monica Polcz, MD:** Conceptualization, Visualization, Methodology, Investigation, Data Curation, Writing – Original Draft, Review & Editing **Jessica Huston, MD:** Conceptualization, Visualization, Methodology, Investigation, Data Curation, Writing – Original Draft, Review & Editing **Meghan Breed, MD:** Methodology, Investigation, Data Curation, Writing – Original Draft, Review & Editing **Marisa Case, RN:** Investigation, Data Curation **Phil Leisy, MD:** Investigation, Writing – Review & Editing **Jeffrey Schmeckpeper, MD:** Investigation, Writing – Review & Editing **Lexie Vaughn, MD:** Investigation, Writing – Review & Editing **Jenna Helmer Sobey, MD**: Investigation, Writing – Review & Editing **Colleen Brophy, MD:** Project administration, Supervision, Writing – Review & Editing **JoAnn Lindenfeld, MD:** Project administration, Supervision, Writing – Review & Editing **Kyle Hocking, PhD:** Conceptualization, Visualization, Methodology, Data Curation, Writing –Review & Editing, Supervision, Funding acquisition **Bret Alvis, MD:** Conceptualization, Visualization, Methodology, Investigation, Data Curation, Writing – Original Draft, Review & Editing, Supervision, Project administration, Funding acquisition.

## Declaration of competing interest

The authors declare the following financial interests/personal relationships which may be considered as potential competing interests:

The authors report no relationships that could be construed as a conflict of interest to the details of this study. Kyle Hocking, PhD, is Founder, CEO and President of VoluMetrix and an inventor on intellectual property in the field of venous waveform analysis assigned to Vanderbilt and licensed to VoluMetrix. Colleen Brophy, MD, is Founder and CMO of VoluMetrix and an inventor on intellectual property in the field of venous waveform analysis assigned to Vanderbilt and licensed to VoluMetrix. Bret Alvis, MD, CSO and is an inventor on intellectual property in the field of venous waveform analysis assigned to Vanderbilt and licensed to VoluMetrix and is married to the COO of VoluMetrix. The remaining authors have no disclosures to report.
