# Supplementary material for: Comparison of clinical symptoms and bioimpedance to pulmonary capillary wedge pressure in heart failure
Source: Am Heart J Plus. 2022 Apr 20;15:100133. doi: 10.1016/j.ahjo.2022.100133 (PMC9119644; doi:10.1016/j.ahjo.2022.100133)
Supplement: Supplemental Table 1 — Utility of the Clinical Findings in Detecting PCWP >22 mmHg. Abbreviation: LR = likelihood ratio; OR = odds ratio; CI = confidence interval. [file mmc1.docx]

**Supplemental Table 1.** Utility of the Clinical Findings in Detecting PCWP >22mmHg. Abbreviation: LR= likelihood ratio; OR= odds ratio; CI= confidence interval.
